# Genetic analysis and gene mapping of a low stigma exposed mutant gene by high-throughput sequencing

**DOI:** 10.1371/journal.pone.0186942

**Published:** 2018-01-03

**Authors:** Xiao Ma, Zhuo Zheng, Fanshu Lin, Tingting Ge, Huimin Sun

**Affiliations:** College of Life Science, Jinggangshan University, Ji’ an, China; Kyung Hee Univeristy, REPUBLIC OF KOREA

## Abstract

Rice is one of the main food crops and several studies have examined the molecular mechanism of the exposure of the rice plant stigma. The improvement in the exposure of the stigma in female parent hybrid combinations can enhance the efficiency of hybrid breeding. In the present study, a mutant plant with low exposed stigma (*lesr*) was discovered among the descendants of the indica thermo-sensitive sterile line 115S. The ES% rate of the mutant decreased by 70.64% compared with the wild type variety. The F_2_ population was established by genetic analysis considering the mutant as the female parent and the restorer line 93S as the male parent. The results indicated a normal F_1_ population, while a clear division was noted for the high and low exposed stigma groups, respectively. This process was possible only by a ES of 25% in the F_2_ population. This was in agreement with the ratio of 3:1, which indicated that the mutant was controlled by a recessive main-effect QTL locus, temporarily named as *LESR*. Genome-wide comparison of the SNP profiles between the early, high and low production bulks were constructed from F_2_ plants using bulked segregant analysis in combination with high-throughput sequencing technology. The results demonstrated that the candidate loci was located on the chromosome 10 of the rice. Following screening of the recombinant rice plants with newly developed molecular markers, the genetic region was narrowed down to 0.25 Mb. This region was flanked by InDel-2 and InDel-2 at the physical location from 13.69 to 13.94 Mb. Within this region, 7 genes indicated base differences between parents. A total of 2 genes exhibited differences at the coding region and upstream of the coding region, respectively. The present study aimed to further clone the *LESR* gene, verify its function and identify the stigma variation.

## Introduction

The exposed stigma of rice is believed to improve the outcrossing rate of the sterile lines [1,2]. The exposed stigma of the rice bypasses the physical barrier of glumes between the foreign pollen and stigma. The stigma is expected to elongatebeyond the glume during exertion. Therefore, a larger area is available for the placement of the pollen to the stigma. Furthermore, the timing that is required to improve the outcrossing rate of the sterile line is an essential parameter that is affected by the exposed stigma. The pollination vigor of the rice stigma will continue for approximately 3 days following flowering. Since the rice stigma remains exerted, the pollination activity is retained. During the next 3 days, the stigma can undergo pollination by receiving foreign pollen. In case of incomplete stigma pollination at the same day, the process is continued, by extending the pollination time, improving the rate of pollination and increasing the seed production of the hybrid rice [3].

However, it is of considerable interest to improve the outcrossing rate of the sterile line in order to enhance the application of the hybrid rice technology. In addition, the method of pollination of the rice cultivation is mainly attributed to self-pollination. It is difficult for the different rice species to outcross during field plantation [4,5]. Nevertheless, the breeding technique for the 3 lines of rice that includes the outcrossing process, aids the preparation of hybrid species and the preservation of male sterile lines. The identification of the exposed stigma of the male sterile line in rice plays an important role in facilitating the process of outcrossing. Stigma is the vital part of the sex organs of the rice. Furthermore, both pistil and stamen are located within the glumes, rendering the structure of the flower unsuitable for outcrossing. In addition, the possibility that the rice glume pollination will hinder cross pollination is particularly low, which renders this process inconvenient with regard to the traditional breeding. Moreover, previous studies have shown that the main factors responsible for the development of the exposed stigma are the external environment and the rice varieties [6]. The improvement of the exposure rate of the stigma in male sterile rice lines by conventional hybridization requires substantial time periods. Therefore, the successful application of advanced molecular biology techniques can aid the discovery of the relevant regulatory genes and the regulatory mechanisms involved in the improvement of the exposure rate of stigma in rice plants.

During long term breeding, a mutant with an exposed stigma was identified, which was derived from the indica male sterile lines 115S by natural mutation. The stigma exposure rate of the mutant was significantly lower compared with the wild type.Thus,thisplant wasdenotedas *lesr* (low exposure stigma rate mutant). In the present study,we investigatedthestigma exposure rate of the mutant species and formulated the genetic population in order to carry out genetic analysis and gene mapping of the mutant gene. The study aimed to lay the foundation for the production of a novel gene clone.The data are of considerable significance regarding the discovery of the regulatory mechanism of the shape of the exposed stigma.

## Materials and methods

### Materials

The rice stigma mutant *lesr* was derived from natural mutation in a descendant of the thermo-sensitive sterile line 115S, and its main phenotype was the low stigma exposure rate compared with that of the wild type rice. *Lesr* was hybridized with the restorer line 93S in order to produce the genetic groups.115S was a thermo-sensitive sterile line cultivated with C815S (female parent) and Y58S(male parent). 93S wasthe restorer line of 115S.

The aforementioned materials were seeded in the rice breeding center of the Jinggangshan University, at a density of 17cm×22cm of transplants and individual plants. The incubation required conventional field water management and timely control of pests and weeds.

### Investigation of stigma exposure rate

In 2015, the investigation of the exposed stigma of the mutant rice plant was measured as follows:The single and double stigma exposure rates were recorded for the wildtype 115S and 93S following 2d of the initial cross breeding [7]. The phenotypic values were expressed by the following formulas:

$$\mathrm{SSE}(\%)=\frac{Ns}{SNP\times 2}$$

$$\mathrm{DSE}(\%)=\frac{Nd\times 2}{SNP\times 2}$$

$$\mathrm{SE}(\%)=\frac{Ns+Nd\times 2}{SNP\times 2}$$

SSE(%): Single stigma exposure rate;
DSE(%): Double stigma exposure rate;
SE(%): Total stigma exposure rate;
Ns: number of single exposure stigma spikelets per panicle;
Nd: number of double exposure stigma spikelets per panicle;
SNP: spikelets number per panicle.

### Genetic analysis

The cross combination was formulated using the mutant *lesr* as the female parent and the restorer line 93S as the male parent in order to obtain the corresponding F_1_ and F_2_ populations. The segregated populations were seeded in the following year, and the segregation of the mutant species was investigated following 2d. The stigma exposure rates of the F_1_ and F_2_ populations were evaluated using SE% as a unit, which was in turn recorded for the segregation ratio estimation. The chi-square statistical test was conducted in order to evaluate differences between the two different populations.

### High-throughput sequencing

Initially, genome DNA was isolated using the CATB methodfrom fresh leaves of the *lesr*, 93s and F_2_ plants. Subsequently, 2 DNA pools were constructed namely H-pool and L-pool, by mixing an equal amount of DNA extracted from 30 high-exposure stigma rate and 30 low-exposure stigma rate lines, respectively. Finally, pair-end sequencing libraries (read length 100 bp) with insert sizes of approximately 500 bp were prepared for sequencing with an Illumina Genome Analyzer IIx sequencer.

The short reads from H-pool and L-pool were aligned to the Nipponbare reference database in build 5 (http://rapdblegacy.dna.affrc.go.jp/) with the BWA software [8]. SNP-calling was conducted by SAM tools software [9]. The low-quality SNPs with base quality value of lower than 20 (<20) and read depth of lower than 4 (<4) × and/or those with higher than 32 (>32) × coverage from the H-pool sequences, were excluded, due to the possibility of false positive results. This could occur as a result of genomic repeat sequence, sequencing and/or alignment errors [10].

### Sequence clustering and association analysis

A total of 2 parameters namely, SNP-index and Δ (SNP-index) were calculated in order to identify candidate regions for the process of early QTL flowering [11]. A SNP-indexis the proportion of reads harboring the SNP that are different from the reference sequence. Δ (SNP-index) was obtained by subtraction of the SNP-index of the H-pool from thatof the L-pool. Thus, for SNP-index = 0, the entire short reads contain genomic fragments from Nipponbare, whereas for SNP-index = 1 all the short reads were from Muromskij. An average ofSNP-index of SNPs that were located within a given genomic interval was calculated using a sliding window analysis of 1 Mb window size and 10 kb increment. The SNP-index graphsfor the H- and L-pools, as well as the corresponding Δ (SNP-index) graph were plotted. Thus, the SNP-index was equal to 0 if all short reads contained genomic fragments from 93S, while the SNP index was equal to 1 if all short reads were from *Lesr*. The SNP-index graphs for the H- and L-pools, and the correspondingΔ (SNP-index), were plotted. The Δ (SNP-index) value was adjusted not to be significantly different from 0 in a genomic region that contained no major QTL of the target gene. The statistical confidence intervals of Δ (SNP-index) for all the SNP positions were estimated with read depths under the null hypothesis of no QTLs. The confidence intervals were plotted along with the Δ (SNP-index). For each read depth, the 95% confidence intervals of Δ (SNP-index) were obtained as described by the study of Takagi [12].

### Marker development and recombinant plant screening

The SSR markers in the predicted region of chromosome 10 of the rice were employed for polymorphism screening between the two parental lines, and between the H- and L-pools. Indel (insertion or deletion) markers were identified by Illumina reads between parents.The BWA/SAM software was used in order to develop additional polymorphic markers between *lesr* and 93S. The primers for the Indel markers were designed with Primer 5 (http://www.PromerBiosoft.com). Following PCR, certain differences in the sequences between the two varieties were observed. SSR and InDel markers were developed in order to screen the recombinant plants and to map the locus of the mutant gene in *lesr*.

### The fine mapping strategy

We investigated the genotypes of all the recessive F_2_ plants using polymorphic markers. The differences noted between the genotypes were assessed using polymorphic markers. The comparison of the genotype region of the parent plants with the phenotypes of multiple recombinant mutants enabled us to limit the location of the region of the mutant gene. This mapping strategy was based on the recombinant-derived progeny and effectively minimized the experimental errors that may have resulted from the genetic background noise [13].

## Results

### Phenotyping for the quantitation of the stigma exposure rate

**Fig 1:**
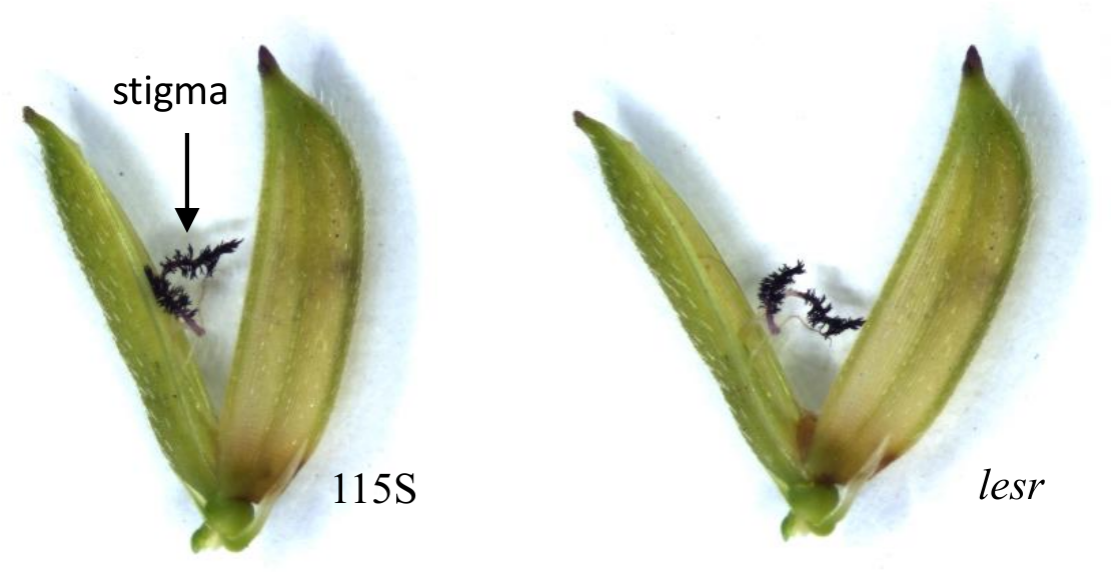
The stigma phenotype of *lesr* mutant.

The mutant *lesr*exhibited no apparent difference in the stigma sizecompared with wild type rice (Fig 1). However,the values of the parameters SSE, DSE and SEthat corresponded to *lesr*were 9.19%,3.34% and 12.53%, respectively (Table 1). The stigma exposure rates of the mutant plants were all reduced to a percentage of 70.36%and/or higher compared with the wild type 115S. The differences noted were significant. The stigma exposure rate of the 93s plant was slightly lower compared with that of the 115s,although the difference was not significant. The 115s plant could be included in the high stigma exposed lines,whereas the stigma exposure rate of the 93s plant was significantly higher compared with that of the mutant *lesr*. Consequently, the 93s plant could be used for geneticcrossing with *lesr*.

**Table 1:** Phenotypic values of stigma exertion.

Trait	Materials		
	lesr	115S	93S
SSE(%)	9.19±2.56a	31.01±4.50b	28.31±3.98b
DSE(%)	3.34±1.48a	11.67±2.06b	10.81±3.02b
SE(%)	12.53±2.44a	42.68±3.39b	39.12±3.48b

Different lower letters indicate significant difference (P <0.05)

### Genetic analysis of mutant characters

**Fig 2:**
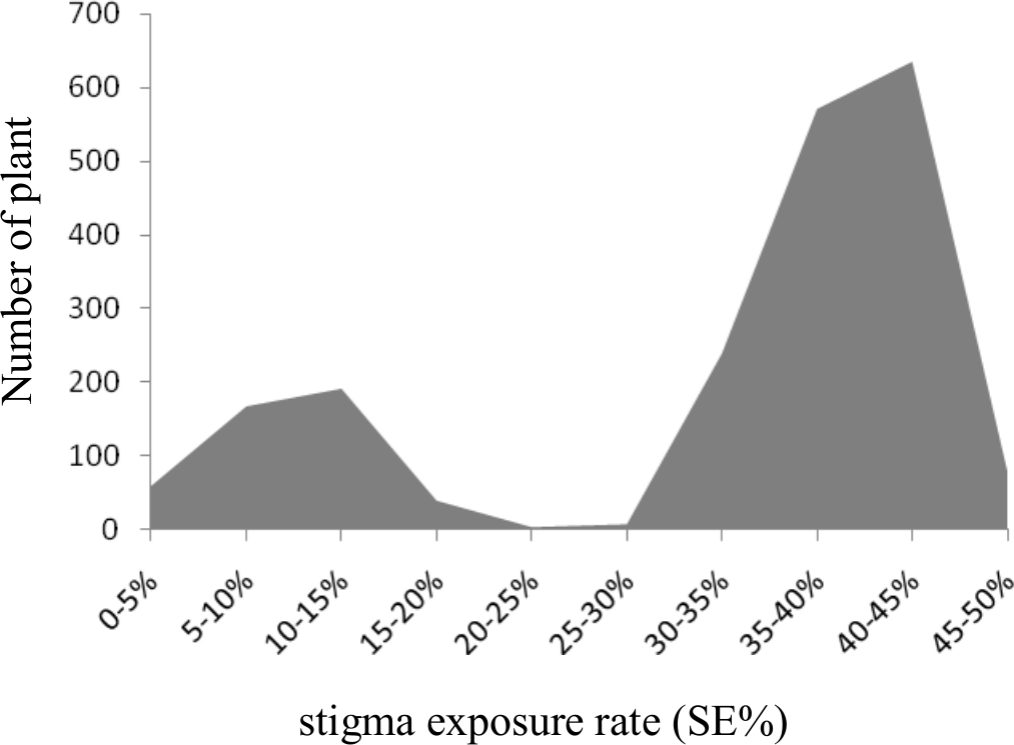
Distribution of the stigma exposure rate in the F_2_ population.

The F_1_ and F_2_ populations were derived by hybridization of mutants and the rice restorer line 93S. All of the F_1_ plants were characterized bya high stigma exposed phenotype, while in the F_2_ population, a clear distinction of the high stigma and low stigma exposed groups was evident by the parameter SE% = 25% (Fig 2). Statistical analysis was conducted by the chi-square test. The results indicated that in 2,000 lines of the F_2_ population, the numbers of the high and low stigma exposure plants were 1,536 and 464, respectively, whereas the separation ratio was 1.00:0.30, with a χ^2^ value of 1.77(χ^2^_0.05,1_ = 3.8) that was in line with the separation of 3:1 ratio. This finding indicated that the mutant is controlled by a recessive QTL loci effect that was temporarily denoted as *LESR*.

### Sequencing quality analysis

Sequencing was conducted on the two parental plants and two bulks with 30 high and/or low exposure stigma rate plants from the F_2_ population, respectively. The SLAF-seq data analysis that generated a genetic region of 34.90 Gb, included 6.89 Gb, 7.32 Gb, 10.75 Gb and 9.94 Gb regions for the 93 S, *lesr*,H-pool and L-pool, respectively. The Q20 ratio was higher than 97.28% (> 97.28%). The GC content ranged from 43.24 to 43.95%. The reference genome size was 374,424 and 240 bp, respectively.All the samples that revealed a mapping rate in the range of 96.32% and 96.41%, exhibited an average cover depth (excluding N area) between 15.77 X to 24.94 X. 1 X coverage (at least one base covered) was noted for more than 92.67% of the plants (Table 2) (https://www.ncbi.nlm.nih.gov/Traces/study/?acc=SRP113583). The data demonstrated that the mapping could be used for subsequent mutation detection analysis based on the data quantity, and the sequencing quality qualification.

**Table 2:** Sequencing data quality.

Sample	Raw Base(bp)	Clean Base(bp)	Q20(%)	GC content(%)	Mapped reads	Total reads	Mapping rate(%)	Average depth(X)	Coverage at least 1X(%)
93S	6,921,873,600	6,888,652,200	98.17	43.95	44,273,829	45,924,348	96.41	15.77	92.67
lesr	7,400,955,900	7,323,906,600	97.28	43.95	47,061,815	48,826,044	96.39	16.25	93.07
H-pool	10,837,182,600	10,751,201,100	97.55	43.34	69,039,249	71,674,674	96.32	24.94	93.79
L-pool	10,040,513,100	9,942,038,100	97.51	43.24	63,865,084	66,280,254	96.36	23.19	93.60

#### Association analysis

**Fig 3:**
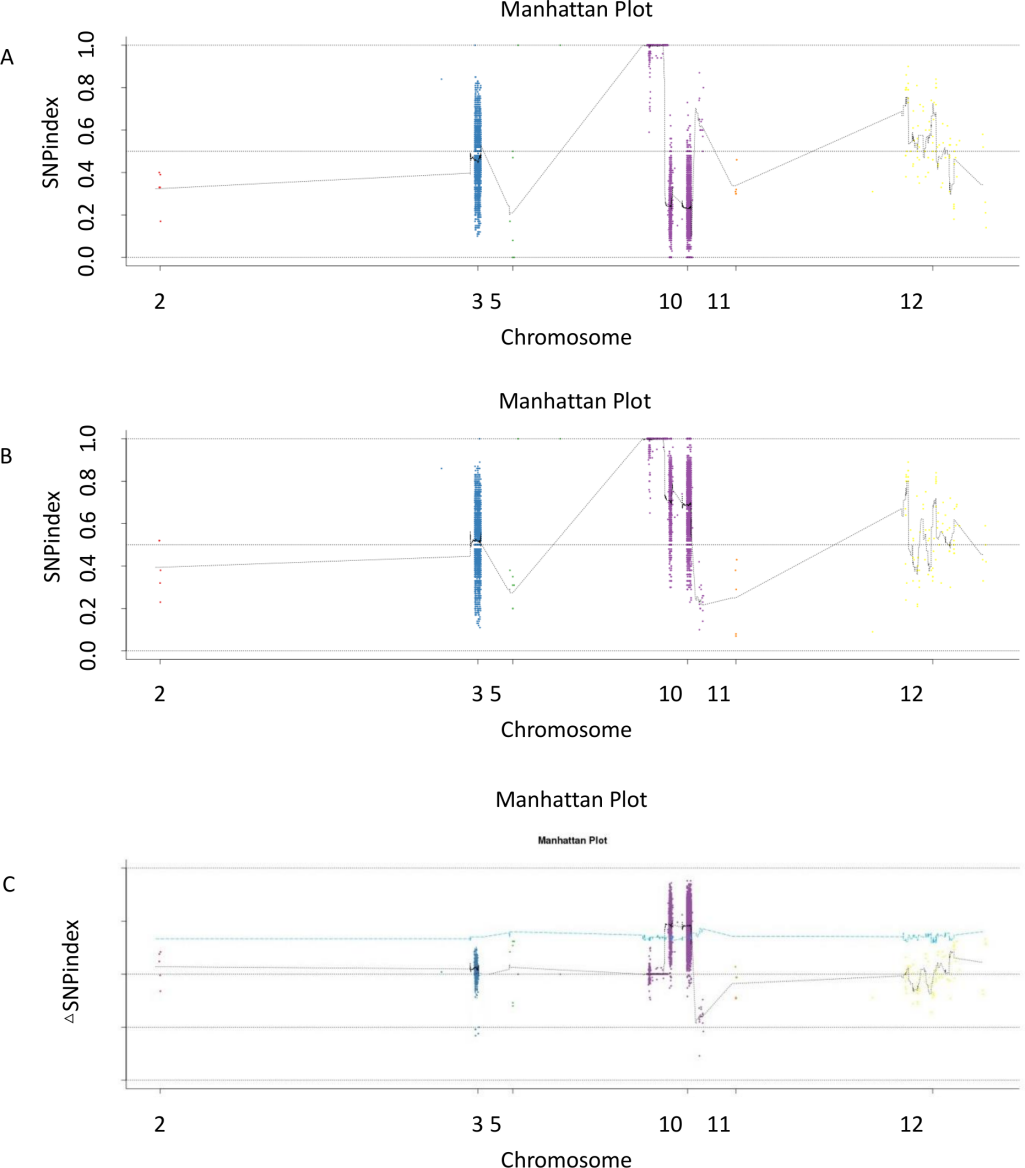
**SNP-index graphs of H-pool (A), L-pool (B) and Δ(SNPindex) graph (C) fromSLAF-seq analysis.** X-axis represents the position of 12 rice chromosomes and Y-axis represents the SNP-index. A candidate gene (*LESR*) location was identified in rice chromosome10 (8.61 to 9.69 Mb or 12.76 to 15.35 Mb) with the criteria that A the SNP-index in H-pool was near 0, B SNP-index in L-pool was near 1, and C theΔ(SNP-index) was over the confidence value (P < 0.05).

The SNP-index was calculated for each SNP identified. The SNP-index graphs were generated for the H-pool (Fig 3A) and the L-pool (Fig 3B) by plotting the average SNP-indexagainst the position of each sliding window in the Nipponbare genome assembly. The Δ SNP-index was calculated by combining the SNP-index in the H-pool and L-pool. The data were plotted against the genome positions (Fig 3C). It was expected that the SNP-index graphical representations of the H and L-pools would be identical for the genomicregions that are irrelevant to the phenotypic difference, whereas the genomic region(s) harboring the mutantgene would exhibit unequal contributions from the 93S and *lesr* parentalgenomes. In addition, the SNP-indices of these regions for the H and S-pools would appear as mirror images [12].

The calculation of the Δ SNPS-index of the two F_2_ population pools was conducted by the following subtraction: SNP-index (B) extreme characters–SNP-index extreme characters (A). The 1,000 displacement test selected 95% confidence level (in blue) as a threshold for the screening process. The ΔSNP-index value was larger than the 95% confidence interval level, if a genomic region harbored a major QTL of the target genes. In the present study, the Δ SNP-index values of the 2 regions were higher than those of the threshold at the 95% significance level. The 2 regions were found on Chr10 from 8.61 to 9.69 Mb and 12.76 to 15.35 Mb, respectively. The analysis was based on the Nipponbare reference database (Fig 3C; Table 2). The data demonstrated that a major mutant QTL/gene was present at the 8.61–9.69 Mb and/or the 12.76–15.35Mb regions on chromosome 10 of the rice plants.

### Development of SSR and Indel markers for mapping the region of LESR

In order to further determine the positioning range of *LESR*, 4 SSR markers were examined in the 8.61 to 9.69 Mb and/or the 12.76 to 15.35 Mb regions on chromosome 10, respectively. The results indicated that the following polymorphic loci: RM25179 and RM25192 (8.61–9.69 Mb) and RM6124 and RM6868 (12.76–15.35 Mb).The polymorphisms were present between the *lesr* and 93S varieties. Following this analysis, the four markers were used to detect the recessive population in the F_2_ population, of which the crossover at RM25179 was detected in 93 plants (79 single crossover plants and 14 double crossover plants). This process was converted to a genetic distance of 11.53 cM. The crossover at RM25192 was detected in 84 plants (71 single crossover plants and 13 double crossover plants), which was converted to a genetic distance of 10.45 cM. The results indicated apparent linkage between the two markers (RM25179&RM25192) and the mutant gene.

Furthermore, crossovers at RM6124 and RM6868 were detected in 11 (11 single crossover plants) and 27 plants (24 single crossover plants and 3 double crossover plants), which were converted to genetic distances of 1.19 and 3.23 cM, respectively. The results further indicated apparent linkage between the two markers (RM6124&RM6868) and the mutant gene,although the genetic distances of RM6124 and RM6868 were lower. This indicated that the chain relationship was closer, enabling the mutation in the RM6124 and RM6868 markers (12.76–15.35 Mb).

**Fig 4:**
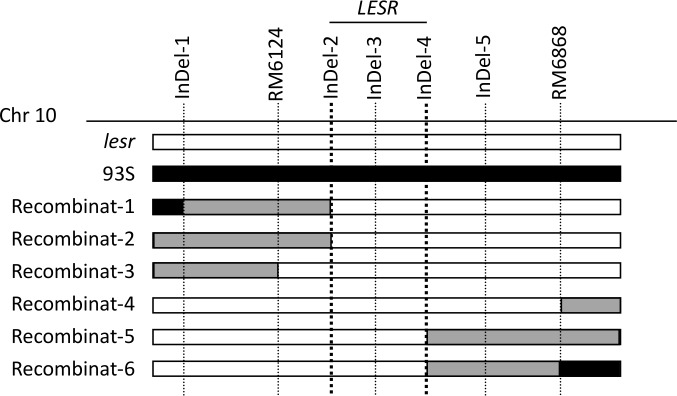
Examination of recombinants in the refined lacation of *LESR*. The black bar is the double crossover plants,the grey bar is the single crossover plants, andthe white bar is the mutant type plants. Seven markers are localized on the physical map. Six recombinants were detected in the region defined by the seven polymorphic markers.Recombinant-1 was from double crossover in the upstream of InDel-1,and from single crossover between InDel-1 and InDel-2; Recombinant-2, Recombinant-3, recombinant-5 were from single crossover between InDel-1 and InDel-2, InDel-1 and RM6124,InDel-4 and RM6868, respectively; Recombinant-4 was from single crossover in the downstream of RM6868;Recombinant-6 were from single crossover between InDel-4 and RM6868, and from double crossover in the downstream of RM6868.Together, these recombinants indicatedthat the *LESR* locus was located within an interval of 0.25 Mb on chromosome 10 flanked by InDel-2 (13.69 Mb) and InDel-4 (13.94 Mb).

The additional design of the 8 InDel markers, aided the examination of the potential polymorphic sites between *lesr* and 93S (the markers are shown in Table 3). The aforementioned primers were used to amplify DNA from the recombinant plant and the two parent plants in order to identify the position of the genetic recombination. A total of six recombinants were recovered within the region defined by the seven polymorphic markers.With the exception of InDel-1 and RM6868, these recombinant plants were products of single-crossovers, while no recombinantion was detected using InDel-3. Taken collectively, the mapping data indicated that the *LESR* locus was located within an interval of 0.25 Mb on chromosome 10 flanked by InDel-2 (13.69 Mb) and InDel-4 (13.94 Mb) (Fig 4, S1 Table). Within this region, 7 genes exhibited base differences between the two parents, of which twogenes (OS10g0404566, OS10g0407000) differed betweenthe mutant and the wild type plants.The differences in the localization intervalamong93S,*lesr* and 115Sand the validation of the markers of the candidate genesare shown in Table 4.

**Table 3:** Polymorphism markers used for genetic mapping of *LESR*.

Marker	Forward Primer	Reverse Primer	Position
InDel-1	ACCGTTCGTCTTATTCAAAA	ACCGTTCGTCTTATTCAAAA	12,973,374
RM6124	TCACGAAGGTGAGACTGACG	CCCATGAATCACCACCACC	13,525,908
InDel-2	GAACCACAAAACCAGACATT	TCAAAAATGCTGACTAGGCT	13,690,353
InDel-3	AATTTATGGCTATGTGTCGG	GCTAAACACTGCTTCGTTTT	13,814,432
InDel-4	TGTCCGAGTTTCTTCATTTT	GGGAGTACACCATCTTTCAA	13,939,718
InDel-5	CTGGAGGAACTGTCTCATTC	CATGCCTAGGCCTATCACTA	14,143,271
RM6868	TGAACATGCCGAGGAAGC	ATATAGAACCCAAAGCCCCC	14,363,911

**Table 4:** The difference between 93S and *lesr* in the localization interval and validation of candidate genes' markers.

No.	Gene ID	Position	93S	*lesr*	115S	Variant	Annotation	Validation markers	
								Forward Primer	Reverse Primer
1	OS10g0403400	13691833	C	T	T	upstream	n/a	AGTTTTCAAGTTTGGTGGGG	CATCAACAGCGGGCGGTG
2	OS10g0403700	13707487	A	G	G	upstream	uncharacterized oxidoreductase	TTTGTTTGAGGAAGACTGG	GAGGAGGGCCTCGTAGGAGCCGTGC
3	OS10g0404566	13784059	C	A	C	upstream;downstream	sucrose transporter	GATAGTGTTAATACTTTTCGAG	GCCGCCATGGCGACGCACACGACGG
		13784298	A	G	G	upstream;downstream			
4	OS10g0406400	13897237	A	G	G	nonsynonymous(Asn→Asp)	mannan synthase	TTGTCATCCCATTGTCTG	TCAATTAGAAATATACACATCTCTA
5	OS10g0406600	13905957	T	G	G	upstream	LYR motif containing protein	TCCGTAGGACAAGCGTGGTT	ATCCAGATAGCTGCTTGGGCTAATG
6	OS10g0406800	13919474	G	A	A	upstream	expressed protein	TCCAACAATTTCCCCTCC	AGCCAGCGGTGAAGACGTACTCCCT
		13919498	A	G	G	upstream			
7	OS10g0407000	13935368	T	G	G	upstream	Pectinesterase	GTTCATCCCTCCTCCCTA	ATATGATGTTGCCGTGTT
		13935387	G	A	G	upstream			
		13935422	A	G	G	upstream			

## Discussion

Rice stigma exertion refers to the phenomenon of stigma exposure following the spikelet’s lemma and palea closure. The degree of exertion of the rice stigma is characterized by the stigma exertion rate, according to the number of the exposed stigma of the spikelet. The stigma exertion rate can be divided into stigma single exposure rate and stigma double exposure rate. The quantifiable indicator of stigma exertion rate is the basic parameter that has been investigated in research studies that examined stigma exposure. Yan [7] reported a positive correlation between stigma single, double and total exertion rates, although the correlation between stigma total exertion rate and the length of the glumes reached a significant level. Therefore, the present study carried out phenotypic identification using the parameter stigma total exertion rate in order to describe the degree of exertion of the stigma. The results demonstrated that the high and low stigma exertion groups could be clearly divided by the border of ES% = 25% in the F_2_ population.

Based on linkage analysis, previous research groups have used different primary genetic segregation populations, namely, F_2_、BC_1_、DH and RILs in order to demonstrate an estimate of the stigma exertion rate [14,15]. In addition, certain studies have examined the position of the stigma exertion rate based on BSA and association analysis. The number of QTLs positioned was very large and widely distributed, whereas the single explained phenotypic variation was estimated at 15%, which indicated that the stigma exertion rate was a quantitative parameter that was influenced by multiple factors [16]. In the present study, the mutant *Lesr* plant was used as the test group and the F_2_ population that produced the hybridized form with the restorer rice 93S, as the genetic population. Using genetic analysis we demonstrated that the degree of the stigma exertion in the F_2_ population exhibited apparent segregation of the plant characteristics, whereas the ratios of the high and low stigma exertion groups were in agreement with the separation ratio of 3:1. The data indicated that this mutant plant type was controlled by a recessive main-effect QTL locus (*LESR*). Consequently, the control of the gene over the degree of the stigma exertion was considerably higher than that noted in previous reports.

The recent development of molecular biology has enabled the studies of specific genes responsible for the exposure of the rice stigma. At least 12 genes have been accurately mapped on the chromosomes of the rice genome, which are mainly distributed on chromosomes 1, 2, 3, 6 and 7. The genes are as follows: *Msp1* [17], *RTS* [18], *ABI5* [19], *ABA8ox1* [20], *TDR* [21], *OsMADS1* [22], *AID1* [23], *FON1* [24], *OsMADS50* [6], *SNB* [25], *UDT1* [26] and *YAB1* [27].

In the present study, we successfully mapped genes related to low rate of stigma exposure on chromosome 10 using bulked segregant analysis in combination with high‑throughput sequencing technology. Following the screening of the recombinant plants with newly developed molecular markers, the genetic region was mapped within 0.25 Mb. Relevant genes on the aforementioned genetic region have not yet been reported. Thus, we propose that *LESR*is a novel gene, which is not allelic with regard to the exposure of the stigma-associated genes that have been previously mapped. Within this genetic region, 7 genes exhibited base differences between the two parents, of which one was at the coding region and the other was upstream of the coding region. Theannotation function related to uncharacterized oxidoreductase, sucrose transporter and mannan synthase enzymes. It is worth noting that there were two genes (OS10g0404566, OS10g0407000) that were different between mutant and wild type plants. Theannotation function revealed the enzymessucrose transporter and pectinesterase respectively.These two candidate genes are highly likely to be the target genes for *LSER*. Additional methods of transgenic validation and gene expression analysis will be employed in future studies in order to further determine the candidate genes and their associated mechanism with regard to the rice stigma exposure.

## Supporting information

S1 Table Segregation of markers in F_2_ recessive mapping population. A: the mutant type genotype plants; B: the double crossover genotype plants; H: the single crossover genotype plants.
(XLSX)
